# Prevalence and molecular characteristics of *Staphylococcus aureus*, including methicillin resistant strains, isolated from bulk can milk and raw milk products in pastoral communities of South-West Uganda

**DOI:** 10.1186/s12879-017-2524-4

**Published:** 2017-06-13

**Authors:** Benon B. Asiimwe, Rossella Baldan, Alberto Trovato, Daniela M. Cirillo

**Affiliations:** 1 Emerging Bacterial Pathogens Unit, IRCCS, Via Olgettina 58, Milan, Italy; 2 Universita Vita-Salute San Raffaele, Via Olgettina 58, Milan, Italy; 3 Department of Medical Microbiology, Makerere University College of Health Sciences, P O Box 7072, Kampala, Uganda

## Abstract

**Background:**

*Staphylococcus aureus* strains are now regarded as zoonotic agents. In pastoral settings where human-animal interaction is intimate, multi-drug resistant microorganisms have become an emerging zoonotic issue of public health concern. The study of *S. aureus* prevalence, antimicrobial resistance and clonal lineages in humans, animals and food in African settings has great relevance, taking into consideration the high diversity of ethnicities, cultures and food habits that determine the lifestyle of the people. Little is known about milk carriage of methicillin resistant *S. aureus* strains (MRSA) and their virulence factors in Uganda. Here, we present the prevalence of MRSA in bulk can milk and raw milk products in pastoral communities of south-west Uganda. We also present PFGE profiles, *spa*-types, as well as frequency of enterotoxins genes.

**Methods:**

*S. aureus* was identified by the coagulase test, susceptibility testing by the Kirby-Bauer disc diffusion and E-test methods and MRSA by detection of the *mecA* gene and SCC*mec* types. The presence of Panton – Valentine Leucocidin (*PVL*) genes and staphylococcal enterotoxins was determined by PCR, while genotyping was by PFGE and *spa* typing.

**Results:**

*S. aureus* were isolated from 30/148 (20.3%) milk and 11/91(12%) sour milk samples. *mecA* gene carriage, hence MRSA, was detected in 23/41 (56.1%) of the isolates, with 21 of the 23 (91.3%) being SCC*mec* type V; while up to 30/41 (73.2%) of the isolates were resistant to tetracycline. Only five isolates carried the *PVL* virulence gene, while PFGE typing revealed ten clusters (ranging from two seven isolates each) that comprised 83% of the sample, and only eight isolates with unique pulsotypes. The largest PFGE profile (E) consisted of seven isolates while t7753, t1398, and t2112 were the most common *spa*-types. Thirty seven of the 41 strains (90.2%) showed at least one of the eight enterotoxin genes tested, with *sem* 29 (70.7%), *sei* 25 (61%) and s*eg* 21 (51.2%) being the most frequently observed genes.

**Conclusion:**

This is the first study to demonstrate MRSA and enterotoxin genes in raw milk and its products in Uganda. The fact that over 90% of the isolates carried at least one gene encoding enterotoxins shows a high risk of spread of foodborne diseases through milk in this setting.

## Background

*Staphylococcus aureus* strains, especially those resistant to methicillin (methicillin resistant *S. aureus*, MRSA) have been regarded as zoonotic agents and there is growing genuine concern about the likely transmission of MRSA between animals and humans from close interaction or from handling and/or consuming MRSA infected animal products [1–5]. In settings where humans depend on animals and their products for food and livelihood, such as in pastoral Africa, contact is intimate and multi-drug resistant microorganisms have become an emerging veterinary and zoonotic issue of public health concern [6–8]. Additionally, *S. aureus* is known to be the third most reported cause of food-borne diseases in the world [4], culpable for production of staphylococcal enterotoxins that cause food poisoning. Moreover, contaminated milk and milk products have frequently been implicated in staphylococcal food poisoning in different industrialized countries [9]. The study of *S. aureus* prevalence, antimicrobial resistance and clonal lineages in humans, animals and food in African settings has great relevance, taking into consideration the high diversity of ethnicities, cultures and food habits that determine the lifestyle of the people.

The treatment of bacterial infections in Uganda’s health and veterinary care settings is largely empirical with no laboratory confirmation to guide therapy, and antibiotics for both human and veterinary use are readily available over the counter. This continuous use and misuse of the antibiotics has resulted into a surge in multidrug microorganisms, now a growing problem on farms, in health care settings and the community [7, 10, 11]. In Uganda, pastoralist communities are vulnerable to zoonotic infections, including MRSA, not only because of the weak veterinary/public health delivery systems but also due to their cherished way of life (culture) dependent on animals and their products. There is limited data about prevalence and spread of *S. aureus* strains in rural pastoral farm environments in Uganda. Previous studies have reported on bacterial carriage of milk in peri and urban farmers in and around the capital, Kampala [7], while another reported on burden of *S. aureus* infection on dairy farms in the neighboring district of Kiboga [6]. However these studies neither assessed resistance to methicillin nor further characterized the isolates for resistance and virulence markers as well as genetic relatedness and spread of the strains in the study environments.

In Uganda, 92% of the milk is marketed unregulated and in raw form [12]. This practice increases the risk of spread of staphylococcal food poisoning due to poor storage and transportation conditions. *S. aureus* is among the leading causes of foodborne bacterial intoxications worldwide [13, 14], with the most notable virulence factors being the heat-stable enterotoxins that cause the sporadic food-poisoning syndrome or foodborne outbreaks [15]. Staphylococcal enterotoxins (SEs) cause toxic shock-like syndromes and have also been implicated in several allergic and autoimmune diseases, in addition to food poisoning; therefore SEs function not only as potent gastrointestinal toxins but also as superantigens that stimulate non-specific T-cell proliferation [16].

The aim of the present study was to establish the prevalence and distribution of methicillin-susceptible *S. aureus* (MSSA) and MRSA in bulk can milk and raw milk products samples at household level. Furthermore, we tested the susceptibility of the isolates to nine drugs and analyzed the genetic relatedness of the isolates by PFGE and *spa* typing. Additionally, resistance and virulence genes (SCC*mec* and *PVL*) as well as frequency of genes encoding three of the classical staphylococcal enterotoxins (SEs): SEA, SEB and SEC; and five additional enterotoxins: SEG, SEH, SEI, SEL, and SEM were assayed.

## Methods

### Study area

The study was carried out in Sanga and Kanyaryeru sub-counties of Kiruhura district, South Western Uganda. The area is a rangeland populated by agro-pastoralist households whose livelihoods are mainly dependent on consumption and trade of cattle and their products, and the area is part of the cattle corridor and is considered the milk basin of Uganda.

### Study design and sampling

A total of 196 homesteads from the two sub-counties (100 from Kanyaryeru and 96 from Sanga) were randomly recruited and sampled. One pooled (bulk can) milk sample and a raw milk product (cow ghee (butter from milk) or sour (fermented) milk) were collected from each household on consent and when available at the time of visit, from July to August 2013. In Kanyaryeru sub-county, 81 milk samples, 60 ghee samples and 53 sour milk samples were collected; while 67milk samples, 57 ghee samples and 38 sour milk samples were collected from Sanga sub-county. In all, 148 milk samples, 117 ghee samples and 91 sour milk samples were collected for the study. Samples were inoculated into Brain-Heart Infusion (BHI) broth in 15 ml propylene tubes and stored on ice in any case for not more than 12 h before transportation to the Department of Microbiology at Mbarara University of Science and Technology for culture and identification.

### Culture, isolation and identification

Initial bacterial culture and isolation was done according to methods described by Cheesbrough [17], with minor modifications. Briefly, 50 μl of culture broth was inoculated on 5% sheep blood agar medium and incubated for 18 - 20 h at 37 °C. Preliminary identification of the bacteria was carried out based on colony characteristics such as shape, size, color and hemolysis patterns. Isolated suspected of being *S. aureus* were shipped in BHI with 20% glycerol to the Emerging Bacterial Pathogens Unit (EBPU) at San Raffaele Scientific Institute in Milan, Italy, for identification and molecular characterization. At the EBPU, all isolates were sub-cultured on mannitol salt agar (Oxoid Ltd., Hampshire, England), then on blood agar (Becton Dickinson, Heidelberg, Germany), and subjected to the tube coagulase test in rabbit plasma with EDTA (Remel, KS, USA). Only isolates that passed the coagulase test were considered for further characterization. Isolates were also screened for susceptibility to eight antibacterial agents (cefoxitin, tetracycline, gentamycin, ciprofloxacin, rifampicin, erythromycin, clindamycin, Sulfamethoxale/Trimethoprim) on Mueller-Hinton agar (Oxoid Ltd., Hampshire, England) according to Kirby-Bauer disc diffusion method and two (oxacillin and vancomycin) by the E-test (Oxoid Ltd., Hampshire, England). Screen tests were performed in 0.5 McFarland according to EUCAST guidelines (http://www.eucast.org/clinical_breakpoints/).

### DNA extraction, SCC*mec* typing and *PVL* genes detection

Bacterial DNA was prepared from isolated colonies suspended in 500 ml Triton X-100 lysis buffer with 1% Triton and 50 mg/ml lysostaphin, incubated at 37 °C for 1 h, followed by phenol-chloroform extraction. Staphylococcal Cassette Chromosome harboring *mecA* (SCC*mec*) gene multiplex PCR typing was done. This was performed using primers and protocols for SCC*mec* types and subtypes I, II, III, IVa, IVb, IVc, IVd, and V and the *mecA* gene according to Zhang et al.[18]. We also assayed for the recently described *mecA* homologue, *mecC* shown to be common in animal-derived strains, using primers and conditions as described in Paterson et al. [19]. The MRSA strain COL (SCC*mec* type I, *mec* gene complex B and *ccr* gene complex 1) was also used as positive control. We further used control strains for SCCmec II (Mu50), III (ANS46), IVa (USA300-FPR3757), IVb (strain from Ospedale San Raffale (OSR) MRSA 864), IVc (strain from OSR collection, MRSA 111), IVd (strain from OSR collection, MRSA 1040) and V (S0385). Unfortunately a control for SCCmec type IV from subtype “e” to subtype “k” was not available to us. Additionally, amplification of the Panton – Valentine Leucocidin (*PVL*) toxin genes, *lukS-PV* and *lukF-PV,* was performed using the *S. aureus* strain ATCC49775 as positive control, and primers and protocols as described by Lina et al. [20].

### Pulsed-field gel electrophoresis (PFGE) and *spa* typing

All isolates were subjected to molecular epidemiological analysis by PFGE after *Sma*I digestion using the CHEF Genomic DNA Plug kit (Bio-Rad) according to a standardized protocol [21]. PFGE was run using a CHEF DRIII system (Bio-Rad). The InfoQuest FP (v5) software (Bio-Rad Laboratories) was used to analyze PFGE profiles, according to interpretation criteria described by Tenover et al. [22]. Clustering analysis was achieved using Dice similarity coefficients and the unweighted pair group method with averages (UPGMA) at 1.5% optimization and 1.5% position tolerance. Additionally, isolates were typed based on sequencing of the hypervariable region of the *S. aureus* protein A gene (*spa*); *spa* types were assigned MLST sequence types (ST) inferred on the Ridom *spa*Server (http://*spa*server.ridom.de) curated by the SeqNet.org initiative [23].

### Staphylococcal enterotoxins genes detection by PCR

DNA amplification was performed using the following conditions: initial denaturation for 15 min at 95^0^ C followed by 35 cycles of denaturation (94^0^ C for 1 min), annealing, and extension (72^0^ C for 1 min), and a final extension step (72^0^ C for 10 min) after the completion of the cycles. The primer sequences, annealing temperatures and expected amplicon sizes are as described in Jarraud et al. [24] and Diep et al. [25]. DNA extracted from the standard strains *S. aureus* Mu50 [26] was used as positive control for enterotoxins SEA, SEC, SEG, SEI, SEL and SEM; while DNA from *S.aureus* COL [27] controlled for enterotoxins SEB and SEH.

## Results

### Prevalence of MSSA and MRSA

Of the samples that were collected, only 56 of 148 milk samples, 25 of 91 sour milk samples and one of 117 cow ghee samples gave growth that was suspected to be *S. aureus* based on colony characteristics such as size, shape, color and hemolysis patterns on blood agar. These samples were then sub-cultured and re-identified at the EBPU and only 30 of 56 isolates from milk and 11 of the 25 isolates from sour milk were coagulase positive, while the single isolate from ghee failed the coagulase test. The prevalence of *S. aureus* in the bulk can milk thus was 30/148 (20.3%) while that in the sour milk was 11/91 (12.1%). Isolation of *S. aureus* was unevenly distributed between the two study areas, with 25 isolates from Kanyaryeru and 16 isolates from Sanga.

### *Drug susceptibility*, *mecA*, SCC*mec* and *PVL* gene

Of the 356 samples cultured (148 fresh milk, 91 sour milk and 117 ghee), *S. aureus* was isolated from 30 (53.6%) of milk, 11 (44%) of sour milk and none were recovered from ghee samples. Carriage of the *mecA* gene (hence MRSA) in this study, identified as part of the Staphylococcal Cassette Chromosome harboring *mecA* (SCC*mec*) after multiplex PCR, was 50% (15/30) and 72.7% (8/11) of the milk and sour milk derived isolates respectively. Overall, only five isolates (5/41, 12.2%) carried the *PVL* virulence gene. A majority of the isolates with the *mecA* gene (21/23; 91.3%) were type V. Nosne of the isolates tested carried the *mecA* homologue, *mecC*._._ When screened with cefoxitin, only two of the 23 isolates carrying the *mecA* gene had zones of inhibition ≤22 mm, hence a discrepancy between the phenotypic and genotypic results. The two isolates resistant to cefoxitin also had MICs of oxacillin above the breakpoint of 2 mg/L, hence resistant. There was a high resistance to tetracycline (73.2%), while all isolates were fully susceptible to ciprofloxacin and vancomycin. The susceptibility pattern of the other drugs tested is shown in Table 1.

**Table 1:** Drug susceptibility pattern of the study isolates to eight of the 10 antimicrobial agents tested

Source	*S. aureus*	Drug susceptibility pattern															
		TE		RD		SXT		CIP		E		CN		DA		VA	
		R	S	R	S	R	S	R	S	R	S	R	S	R	S	R	S
Milk	30	20	10	1	29	1	29	0	30	1	29	1	29	2	28	0	30
Sour milk	11	10	1	0	11	1	10	0	11	0	11	0	11	0	11	0	11
Total	41	30	11	1	40	2	39	0	41	1	40	1	40	2	39	0	41

*TE* tetracycline, *RD* rifampicin, *SXT* trimethoprim-sulphamethoxale, *CIP* ciprofloxacin, *E* erythromycin, *CN* gentamycin, *DA* clindamycin, *VA* vancomycin

### PFGE restriction profiles and *spa* types

There were ten clusters (ranging from two to seven isolates each) that comprised 83% of the sample, while only eight isolates had unique pulsotypes (Table 2). The largest PFGE profile (E) consisted of seven isolates, all with unknown *spa* types. The most common *spa* types were t7753 and t1398 (four isolates each), while others were t2112 (three isolates), t3992 and t127 (two isolates each). The PFGE profiles and *spa* types of the isolates are shown in Table 2.

**Table 2:** Molecular characteristics of the 41 isolates of *S. aureus* in the study

Isolate ID	Pulsotype	*spa* type	MLST	PVL	*mec*A	SCCmec
T130 m	A1	t1398		−	−	−
T132 m	A1	t2112		−	+	V
T111 m	A2	t380		+	−	−
T119 m	A3	t7753		−	−	−
T147y	A4	t2112		−	+	V
T067 m	B1	t127	ST-1	−	+	V
T089y	B1	t127	ST-1	−	+	V
T120y	C1	t1236	ST-97	−	−	−
T139 m	C1	t7753		−	−	−
T115 m	C2	t10103		−	−	−
T089 m	C3	t7753		−	−	−
T099y	C4	t14299		−	+	V
T041y	D1	Unknown		−	+	V
T042 m	D2	Unknown		−	−	−
T127 m	D3	Unknown		−	+	V
T031 m	E1	Unknown		−	+	V
T117 m	E2	Unknown		−	−	−
T136 m	E3	Unknown		−	+	V
T105 m	E4	Unknown		−	−	−
T143 m	E4	Unknown		−	−	−
T094 m	E5	Unknown		−	−	−
T148 m	E5	Unknown		−	+	V
T019 m	F1	t645	ST-121	+	+	V
T057 m	F1	Unknown		−	+	V
T103 m	G1	t1398		−	+	V
T145 m	G1	t1398		−	+	V
T030 m	G2	t1398		−	−	−
T098y	H1	t186	ST-88	−	−	−
T120 m	H2	Unknown		−	+	V
T037 m	I1	Unknown		−	+	V
T067y	I2	Unknown		−	+	V
T059y	L1	t2112		+	+	V
T091 m	L2	t1236	ST-97	−	−	−
T032 m	Unique	Unknown		+	+	IV
T037y	Unique	Unknown		−	+	V
T049 m	Unique	Unknown		−	−	−
T055 m	Unique	Unknown		−	−	−
T056 m	Unique	t3992	ST-97	−	+	V
T055y	Unique	t3992	ST-97	−	−	−
T144 m	Unique	t7753		−	+	IVc
T157y	Unique	t3772		+	+	V

PVL = presence (+) or absence (−) of the *PVL* gene; mecA = presence (+) or absence (−) of the *mecA* gene; SCCmec = SCCmec-types according to Zhang et al. [18]

### Frequency of staphylococcal enterotoxins (SEs)

Results for the detection of eight genes encoding the enterotoxins SEA, SEB, SEC, SEG, SEH, SEI, SEL, and SEM are shown in Table 2, in which 37 of 41 strains (90.2%) were positive for at least one enterotoxin gene. The distribution of enterotoxin genes in MRSA and MSSA strains as well as in milk and sour milk samples was unequal, with MSSA and milk-derived isolates showing more strains with multiple enterotoxins. There were 17/30 (56.7%) milk-derived strains carrying at least three enterotoxins genes while only 3/11(27.3%) of isolates from sour milk carried more than three. Among the genes that code for three of the classic enterotoxins assayed (SEA-SEC), *sec* was the most frequent with 10 isolates (24.4%); while in the other enterotoxins, *sem* (29/41; 70.7%), *sei* (25/41; 61%) and s*eg* (21/41; 51.2%) were the most frequently observed genes. The proportion of all the other enterotoxins can be seen in Table 3. Only four isolates (two from raw milk and two from sour milk) did not carry any of the eight enterotoxin genes that were assayed.

**Table 3:** Genotypic profile of *S. aureus* strains, according to *sea*, *seb*, *sec*, *seg*, *seh*, *sei*, *sel,* and *sem* genes

Genotypic profile	Raw milk (*n* = 30)	Sour milk (*n* = 11)	Total (%)
*sea*	1	1	2 (4.9)
*seb*	4	2	6 (14.6)
*Sec*	7	3	10 (24.4)
*Seg*	16	5	21 (51.2)
*She*	13	2	15 (36.6)
*Sei*	17	8	25 (61)
*Sel*	8	2	10 (24.4)
*Sem*	21	8	29 (70.7)
*sea* + *seb* + *sec*	3	0	3 (7.3)
*seg* + *seh* + *sei* + *sel* + *sem*	9	1	10 (24.4)
At least 1 SE gene	28	9	37 (90.2)
No SE gene detected	2	2	4 (9.8)

## Discussion

Pastoralists within Uganda’s cattle corridor are afflicted by a variety of ecosystem-based human/livestock health problems, especially those zoonotic in nature, with potential to spill over to neighboring communities and affecting the tourism industry. Unfortunately, not much has been done to estimate the burden of many such potentially zoonotic infections in these marginalized communities. *S. aureus*, including MRSA, has recently been found to be a zoonotic problem in different settings [2, 3, 28]. We set out to study the prevalence of methicillin resistance, virulence factors, and genetic relatedness of *S. aureus* strains isolated from bulk can milk and milk products from pastoral households around L. Mburo national Park.

*S. aureus* was isolated in 20.3% of bulk can milk samples and 12.1% of sour milk samples. Generally, there is a paucity of data on *S. aureus* from milk and raw milk products in the region. In urban and peri-urban Kampala, a recent study of 97 milk samples yielded 85 pure cultures, but only 1 of 58 (2%) Gram-positive bacteria was identified as *S. aureus* while 20 of 58 (34%) were coagulase negative Staphylococci [7]. Another study carried out in dairy farms in rural Kiboga (another cattle corridor district) in Uganda, however, found that the most prevalent bacterial pathogens associated with sub-clinical mastitis in dairy cattle were Staphylococci such as Coagulase negative Staphylococci and *S. aureus* in 64.42 and 16.56% of the cases respectively [6], comparable to our finding of 19.6% *S. aureus* in fresh bulk can milk samples in the current study. These findings support the expectation that *S. aureus* from infected udders may contaminate bulk milk and, subsequently, raw milk products as has been observed elsewhere [29].

SCC*mec* PCR showed that 23/41 (56.1%) of the isolates carried the *mecA* gene implicated in resistance to methicillin. Elsewhere in Africa, however, low level MRSA carriage in raw milk has been reported in Algeria where only two of five isolates were screened by the disc diffusion test, but there was no testing for the *mecA* gene [30]. In the current study, only two isolates had zones of clearance less than 22 mm around cefoxitin discs as recommended by EUCAST, to be considered MRSA. The two isolates also had MIC of >2 mg/L on oxacillin strips, hence resistant. The discrepancy between the phenotypic and genotypic results seen in this study may be due to a lack of expression of *mecA*. Routine oxacillin tests often fail to detect very heterogeneous MRSA populations, which consequently are considered methicillin-susceptible *S. aureus* (MSSA) because of their usual susceptibility to most non-β-lactam antistaphylococcal antibiotics. Therefore, several parameters have been recommended to improve results and these include increasing the inoculum, growth at a low temperature, an oxacillin screen test with NaCl, or protracted incubation [31]. More recent methods for detection of MRSA include the oxacillin E-test (AB BIODISK, Solna, Sweden) for determination of MICs, and the automated Vitek 2 system (bioMe’rieux, La Balme les Grottes, France). Phenotypic results will therefore vary between laboratories depending on the system used. Detection of the *mecA* gene, therefore, would be a good way of standardizing identification of MRSA across laboratories. The results are similar to those in another study comparing methods for the detection of MRSA on isolates from foods of animal origin in Italy by Corrente et al. [31]. In that study, none of the six MRSA strains identified by *mecA* PCR were detected by the cefoxitin test (sensitivity of 0%), and they concluded that analysis for MRSA in isolates from food of animal origin is better done with the mecA gene-specific PCR rather than conventional phenotypic assays [31]. More studies in human derived strains also render support to low sensitivity of the cefoxitin test. For example, a recent study of low level methicillin resistance in *mecA* positive *S. aureus* strains showed zones of clearance of 28 mm diameter by cefoxitin testing [32]. In the current study, 17 of the 23 *mecA* positive strains also had zones of clearance of ≤28 mm (Table 1). The observation of a high proportion of MRSA in the study sample is very worrisome because studies have shown that MRSA readily jumps from animals to exposed veterinarians, farm works and other farm animals [2, 5, 28]. This, to the best of our knowledge, is the first report of MRSA isolated from milk and raw milk products under communally grazing pastoral households in Uganda. In this setting where animal – human interaction is intimate due to the pastoralists’ cherished way of life, it creates an urgent public health concern.

Genotype analysis was done by SCC*mec* typing and PFGE. Of the 23 *mecA* positive (MRSA) isolates, 21 harboured the SCC*mec* type V. The other SCC*mec* types found can be seen in Table 2. It has recently been noted that community-associated MRSA (CA-MRSA) isolates, which carry SCC*mec* type IV or V, are now prevalent and exceed methicillin-susceptible *S. aureus* (MSSA) in skin and soft tissue infections, and such isolates were reported to cause severe, often necrotizing, soft tissue infections and pneumonia [33, 34]. Their presence in milk creates risk of spread of these genotypes in the communities. Additionally, assaying for the virulence marker *PVL* showed that only four isolates were positive for this gene. A previous study of 50 *S. aureus* isolates from 14 dairy cow herds in Italy identified the *PVL* gene in more than 50% of the isolates [35], while another in Brazil reported that none of the 84 milk derived *S. aureus* isolates harbored the *PVL* gene [36].

According to criteria proposed by Tenover et al., [22], strains that differ by three bands or fewer by PFGE are likely to be closely related. The authors emphasized that these criteria apply to epidemiologically related isolates. By these criteria, isolates in the current study group into ten clusters of two to seven isolates each, and only eight unique strains. Since these were not outbreak strains, *spa* typing was used to determine the clonal lieneages of the isolates. The most common lineages were t7753 and t1398 with four isolates each. In all, there were 13 different lineages, but most of the isolates (17/41, 41.5%) could not be assigned to any lineage and were designated as unknown (Table 2). Only one lineage in the current study, t645, had been isolated from one mastitic cow and four milkmen in peri-urban Kampala [7]. The most common spa types associated with clone ST398 (t011, t034, t108, t567, t571, t588, t753, t753, t779, t898, t899, t943, t1184, t1197, t1254, t1255, t1451, t1456, t1457, t2123, t2287, t2329, t2330, t2383, t2582, t2748, t2971, t2974, t3013, t3014, t3053, t3146, or t3208) according to the recent work [37] were not found among our isolates. However, we detected other spa types that have been reported to be associated with LA-MRSA strains other than ST398 (Table 2).

This, to the best of our knowledge, is the first report of SEs in milk from Uganda. While literature elsewhere suggests that the classical SEA is the most frequently observed in enterotoxigenic strains of *S. aureus* [38], it was found in only two (4.9%) of the strains in the current study. This is probably because of the fact that different foods and strains carry different enterotoxins, as observed elsewhere [39]. On the other hand, the more recently discovered *seg* gene was observed in 21 (51.2%) of 41 isolates, and in 85.7% of the cases, it was associated with *sei*, which was present in 25 (61%) of the isolates, similar to reports in other studies in Brazil [40] and France [41]. These genes are known to be frequently found together because they are within the same cluster, in a 3.2 kb DNA fragment as reported elsewhere [42]. In East Africa, most milk is produced by small holders and the bulk of it (86% in Kenya and 92% in Uganda) is traded through unregulated channels as unpasteurized milk or milk products [12]. Raw and sour milk are consumed in these settings for such beliefs as enhanced nutritional quality and better taste. The fact that over 90% of the isolates in the current study carried at least one gene encoding for enterotoxins shows a high risk of spread of foodborne diseases in this setting, with milk as a vehicle for dispersion. There is a need for farmer education and health promotion messages in the communities about the need for pasteurization of milk as this will kill *S. aureus*, therefore reducing enterotoxin production.

## Conclusion

Our results show a high proportion of MRSA in raw milk and its products in the cattle corridor of Uganda, threatening treatment options for *S. aureus* in the near future in this setting, especially if the strains cross the species barrier into humans. Isolates carrying SCC*mec* type V are wide spread in this setting. The fact that over 90% of the isolates in the current study carried at least one gene encoding for enterotoxins shows a high risk of spread of foodborne diseases. A limitation of this study is that the enterotoxin genes were not studied for expression, therefore we cannot confirm their significance in cases of food poisoning. Additionally, the initial processing and culture technique for the samples was not good enough whereby only 82 of the 356 samples yielded suspected *S. aureus*.
